# Supplementary material for: Universal and Expanded Screening Strategy for Congenital Cytomegalovirus Infection: Is Pool Testing by a Rapid Molecular Test in Saliva a New Choice in Developing Countries?
Source: Viruses. 2024 May 13;16(5):772. doi: 10.3390/v16050772 (PMC11125597; doi:10.3390/v16050772)
Supplement: Supplementary file 1 [file viruses-16-00772-s001.zip › viruses-2985027-supplementary.pptx]

## Slide 1
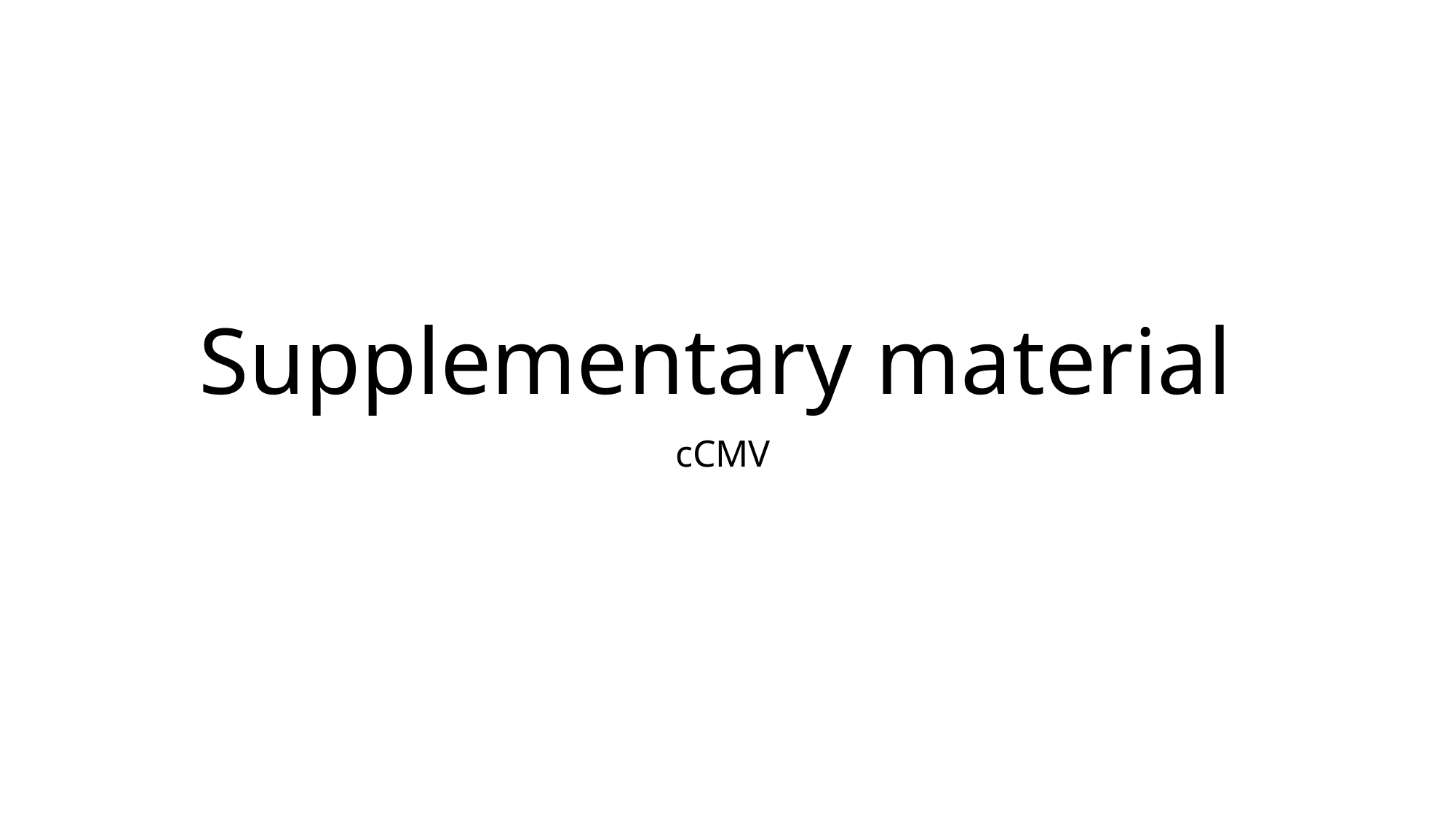

# Supplementary material
cCMV

## Slide 2
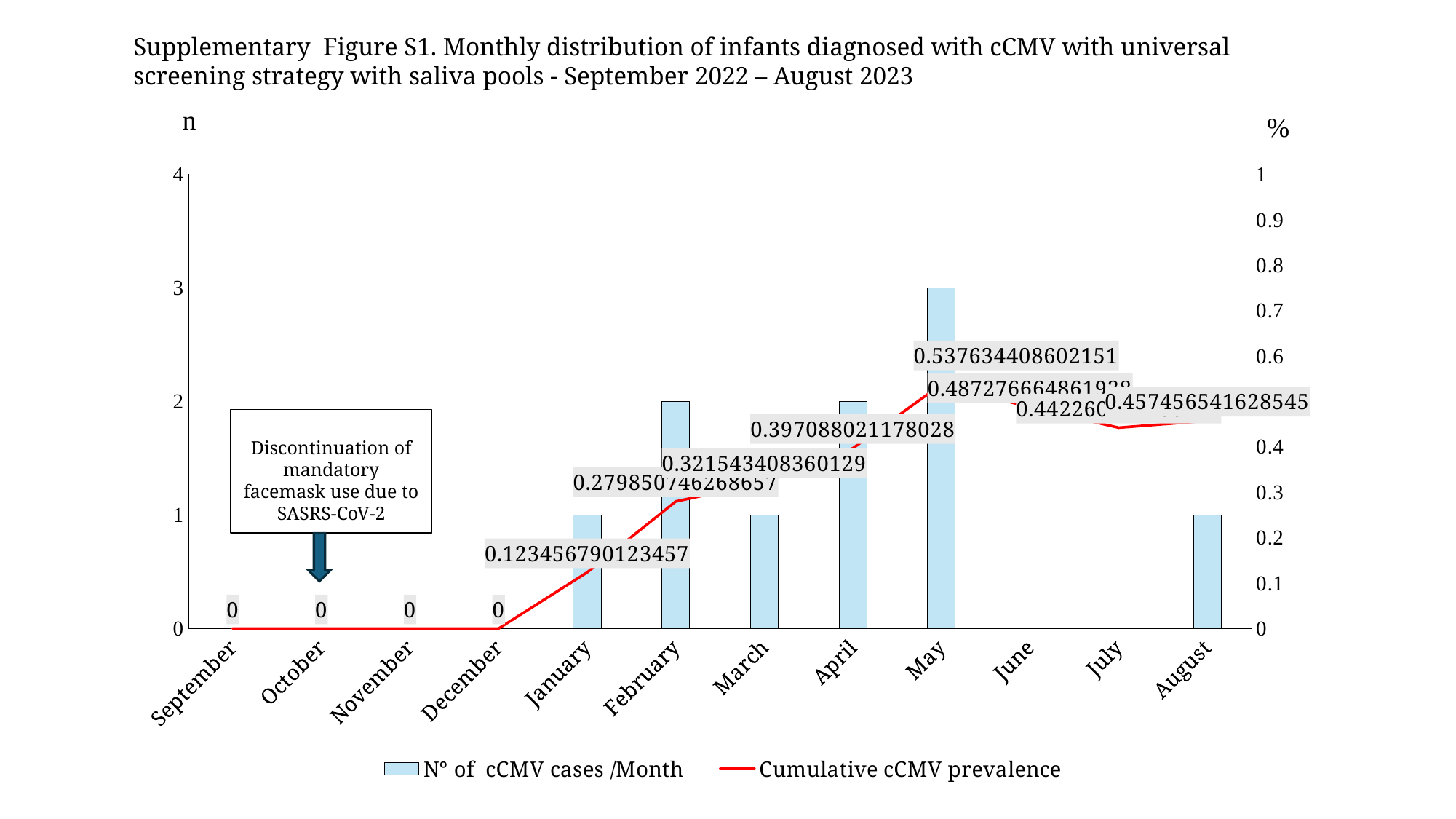

Supplementary Figure S1. Monthly distribution of infants diagnosed with cCMV with universal screening strategy with saliva pools - September 2022 – August 2023
n
%
### Chart
| Category | N° of cCMV cases /Month | Cumulative cCMV prevalence |
|---|---|---|
| September | 0.0 | 0.0 |
| October | 0.0 | 0.0 |
| November | 0.0 | 0.0 |
| December | 0.0 | 0.0 |
| January | 1.0 | 0.12345679012345678 |
| February | 2.0 | 0.2798507462686567 |
| March | 1.0 | 0.3215434083601286 |
| April | 2.0 | 0.3970880211780278 |
| May | 3.0 | 0.5376344086021506 |
| June | 0.0 | 0.48727666486193827 |
| July | 0.0 | 0.4422604422604423 |
| August | 1.0 | 0.4574565416285453 |
Discontinuation of mandatory facemask use due to SASRS-CoV-2

## Slide 3
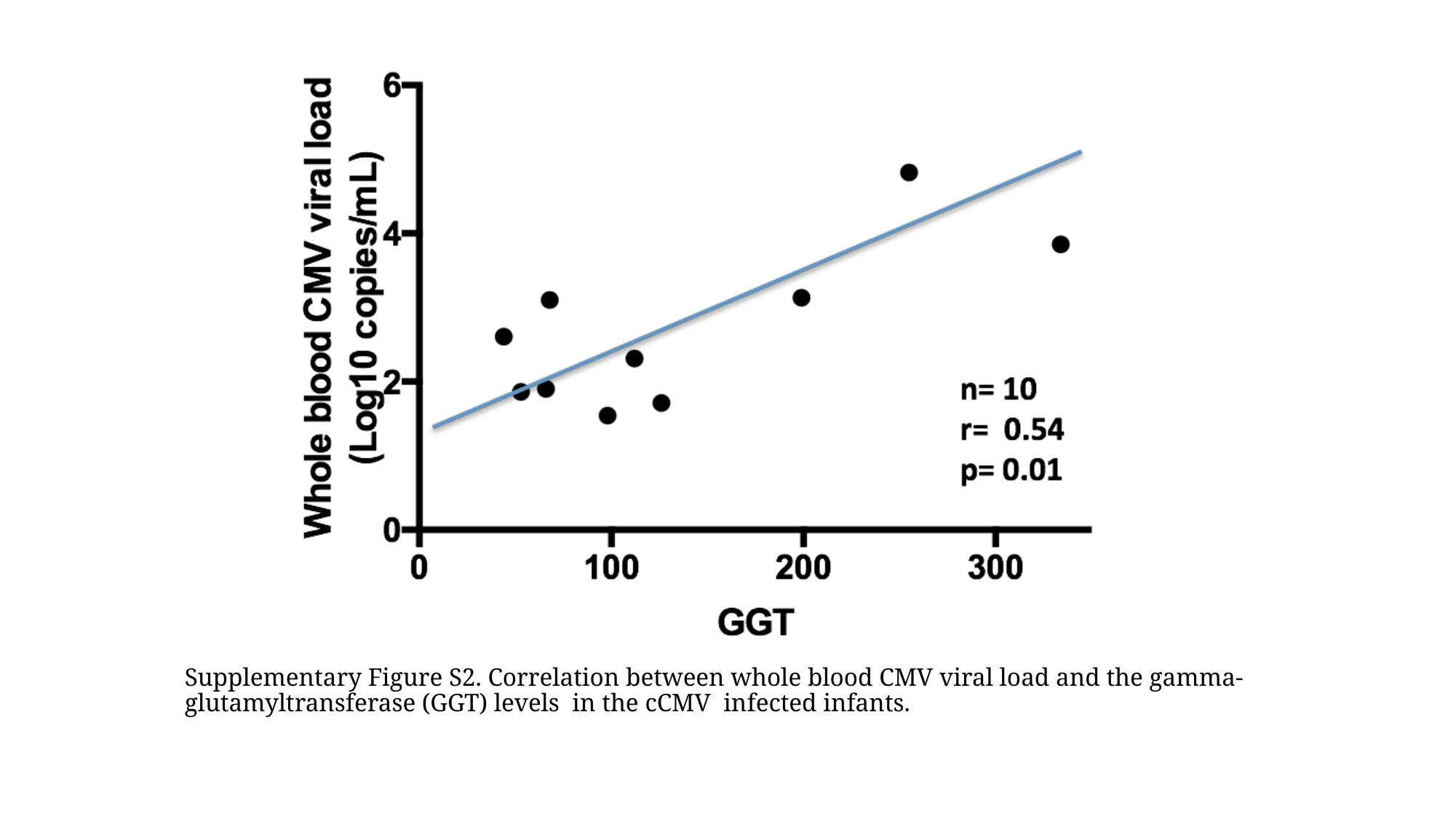

# Supplementary Figure S2. Correlation between whole blood CMV viral load and the gamma-glutamyltransferase (GGT) levels  in the cCMV infected infants.
